# Eyring–Polanyi Rate Theory for the Homogeneous Nucleation of Organic Crystals from Solution

**DOI:** 10.1021/acs.cgd.5c00524

**Published:** 2025-12-12

**Authors:** Sven L. M. Schroeder

**Affiliations:** † School of Chemical and Process Engineering, 4468 University of Leeds, Leeds LS2 9JT, U.K.; ‡ Diamond Light Source, Harwell Science & Innovation Campus, Didcot OX11 0DE, U.K.; § Future Continuous Manufacturing and Advanced Crystallisation (CMAC) Hub, Research Complex at Harwell (RCaH), Rutherford Appleton Laboratory, Didcot OX11 0FA, U.K.

## Abstract

Using elementary concepts of chemical reaction kinetics, the rates of primary homogeneous organic crystal nucleation from supersaturated solutions are modeled as nucleation with first order kinetics from large solute density fluctuations (LSDFs). Solute density fluctuations are modeled as diffusively driven many-body collisions of weakly interacting solvated solute molecules. The first order rate constant is a system-specific supersaturation-independent rate constant for nucleation in LSDFs. It is shown for several solute–solvent systems that the temperature-dependence of this nucleation rate constant exhibits Arrhenius behavior. The activation enthalpy (Δ*H* ^⧧^) and activation entropy (Δ*S* ^⧧^) for homogeneous nucleation is determined from an Eyring–Polanyi analysis of temperature-dependent nucleation rates. The steps of the Eyring–Polanyi analysis are described in detail for the homogeneous nucleation of l-histidine from aqueous solutions. The analysis is also applied to temperature-dependent homogeneous nucleation rates of salicylic acid in four different solvents. For all systems, the supersaturation- and the temperature-dependence of the primary homogeneous nucleation rates are completely reproduced by reference to temperature-dependent solubility data through the activation parameters Δ*H* ^⧧^ and Δ*S* ^⧧^. Δ*H* ^⧧^ is for all examined systems approximately 12 times the enthalpy of solution determined from solubility data, suggesting that nucleation from LSDFs resembles, at the molecular level, a reverse dissolution process. Within the temperature ranges used for measuring nucleation rates, the Gibbs energy of activation Δ*G* ^⧧^ does not vary strongly, resulting in an inverse correlation between enthalpies and entropies of activation. The Eyring–Polanyi framework thus provides, for the first time, a method for semiquantitative predictions of homogeneous nucleation rates from temperature-dependent solubilities.

## Graphical abstract

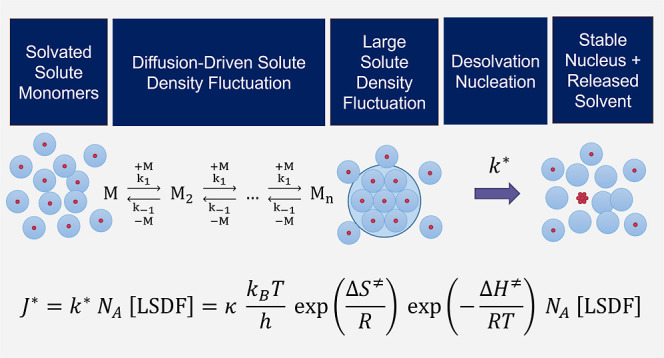

## 1 Introduction

Crystallization from solution is one of the most common naturally occurring phase separation processes and the basis for many economically important manufacturing operations.^1,2^ Despite the importance of crystallization across science and engineering, there is currently no theory that predicts the rates of homogeneous crystal nucleation from solution sufficiently accurately to facilitate a reliable design of solution crystallization processes ab initio or from available solution property data (e.g., solubilities).^2–10^

The primary homogeneous nucleation of crystals from solution is an activated process associated with a free energy barrier ΔG*. The homogeneous nucleation rate *J* is usually expressed as the number of nucleation events per time and volume of solution. Currently, the most widely used framework for analyzing and interpreting homogeneous crystal nucleation rates in solution is classical nucleation theory (CNT),^5–9^ which considers *J* as the product of (i) the number density of solute molecules *C*_0_ (treating every solute molecule as a potential nucleation site), (ii) the rate f* by which other solute molecules attach to a growing nucleus, (iii) the Zeldovich factor *Z*, which describes the probability that a nucleus at the top of the free energy barrier forms a stable crystal, and (iv) an exponential activation term containing ΔG*, the temperature *T* and the Boltzmann constant *k*_B_

1 $$J=f^{*}ZC_{0}\;\exp\left(-\frac{{\Delta G}^{*}}{k_{B}T}\right)$$

The core hypothesis of CNT is that the stability of nuclei is determined by the balance between stabilizing volume and destabilizing surface energy terms so that their size determines the ΔG* barrier. The volume term arises from the cohesive free energy gain associated with the transition of molecules from the solution to the solid phase. The surface term stems from the surface tension γ, i.e., the energy associated with the formation of the solid/solution interface. Very small nuclei are expected to be thermodynamically unstable because of their high surface area, which results in a surface energy penalty that outweighs the cohesive energy gain. For spherical nuclei, the energy balance tips in favor of the stabilizing cohesive energy term for a critical minimum radius *r*_c_ of the nuclei. Supercritical nuclei with radii above *r*_c_ continue to grow to crystals, while subcritical nuclei redissolve into the solution. Overcoming *r*_c_ requires supersaturation of the solution, which decreases the free energy barrier ΔG* by raising the chemical potential of the solute molecules relative to the crystalline nuclei. The supersaturation-dependent form of the nucleation rate eq 1 is^3,4,7,11^

2 $$J=f^{*}\left(S\right)Z\left(S\right)C_{0}\;\exp\left(-\frac{B}{\ln^{2}S}\right)$$

which contains the supersaturation ratio *S* and the parameter *B* that encompasses the influence of surface tension and temperature alongside the molecular volume *v* and a shape factor *c*

3 $$B=\frac{4v^{2}}{27}{\left(\frac{c\gamma }{k_{B}T}\right)}^{3}$$

In writing down eq 2 it has been indicated that the Zeldovich factor *Z* and the attachment rate f* are somewhat dependent on the supersaturation ratio *S* as well.^3^

A fundamental challenge for the analysis of homogeneous nucleation rates by CNT is that the theory depends on a priori unknown parameters whose values are cross-correlated and hence require careful examination of experimental nucleation rates, which are quasi-exponentially increasing as a function of *S*. It was recently noted^12^ that modeling of homogeneous nucleation rates as a first order rate process taking place in diffusively formed regions of high local solute density reproduces the supersaturation-dependence of nucleation rates in terms of a single system-specific empirical parameter, namely a first-order nucleation rate constant k*. The central hypothesis underlying this analysis was that nonideal solutions of solutes can be approximately modeled as an ideal solution of *solvated* solute molecules. The rationale for taking this approach was as follows: in an ideal solution, the enthalpy of mixing is zero because solute–solute, solute–solvent and solvent–solvent interactions in the system are, on average, the same.^13^ In real solutions, most notably those in which solute–solvent interactions create a solvation shell around each solute molecule, this condition is not fulfilled; solvation of solute molecules is associated with nonzero contributions to the mixing enthalpy. However, once solvation has taken place, the interactions of solvated solutes are dominated by those of the solvent molecules in their solvation shell: the solution is then characterized by solvent–solvent, solvated solute–solvated solute, and solvated solute–solvent interactions. All these interactions are interactions between free solvent molecules and/or solvent molecules in the solvation shell, which are broadly similar in character and strength. A real solution can thus be considered akin to an ideal solution of solvated solute molecules. It is of course important for this analysis to identify the solvated solute speciation correctly. For example, in a previously analysis the likely dimerization of benzoic acid and *p*-toluic acid in their toluene solutions was taken into account.^12^

This previous analysis showed how the assumption of an ideal solution of solvated solutes predicts a steady-state size distribution of solution regions with high local solute density, which are formed by diffusively driven multibody collisions of the solvated solute monomers. It was shown that this diffusion-driven pre-equilibrium feeds nucleation with first order kinetics. Conceptually, this model is analogous to the analysis of bimolecular reaction rates in solution^14,15^ as well as the Michaelis–Menten analysis of enzymatically catalyzed biochemical transformations.^16^ They also describe a first order rate process for the formation of a product P from a precursor AB, in which the precursor is supplied by a diffusion-driven pre-equilibrium of monomeric solute species A and B^15^

4 $$A+B\begin{array}{l} \overset{k_{1}}{\to }\\ \underset{k_{-1}}{\leftarrow } \end{array}AB\overset{k_{2}}{\to }P$$

In this equation, *k*_1_, *k*_–1_ and *k*_2_ are the rate constants associated with the three elementary rate processes. With these constants, the rates of formation of AB and P in terms of the concentrations [A], [B], [AB] and [P] of, respectively, the reactants, the precursor and the product, become

5 $$\frac{d\left[AB\right]}{dt}=k_{1}\left[A\right]\left[B\right]-k_{-1}\left[AB\right]-k_{2}\left[AB\right]$$

6 $$\frac{d\left[P\right]}{dt}=k_{2}\left[AB\right]$$

Applying the steady-state approximation to the formation of AB

7 $$\frac{d\left[AB\right]}{dt}\approx 0=k_{1}\left[A\right]\left[B\right]-k_{-1}\left[AB\right]-k_{2}\left[AB\right]$$

the rate of product formation follows as^14,15^

8 $$\frac{d\left[P\right]}{dt}\approx \frac{k_{1}k_{2}}{k_{-1}+k_{2}}\left[A\right]\left[B\right]$$

When the conversion of AB to P is the rate-limiting step, then *k*_–1_ ≫ *k*_2_ and we obtain

9 $$\frac{d\left[P\right]}{dt}\approx \frac{k_{1}}{k_{-1}}k_{2}\left[A\right]\left[B\right]=Kk_{2}\left[A\right]\left[B\right]$$

The equality of the right introduces the thermodynamic equilibrium constant *K* for the pre-equilibrium

10 $$K=\frac{k_{1}}{k_{-1}}$$

For the opposite case, *k*_–1_ ≪ *k*_2_, the rate is determined by the diffusive rate by which A and B encounter each other in solution, and we get

11 $$\frac{d\left[P\right]}{dt}\approx k_{1}\left[A\right]\left[B\right]$$

which may, for example, apply for reactive nucleation processes.

Values for the diffusion rate constant *k*_1_ can be estimated via the Stokes–Einstein equation from the viscosity η of the solution^14^

12 $$k_{1}=\frac{8RT}{3\eta }$$

where *R* is the universal gas constant and *T* the temperature of the solution. Values for *k*_1_ are typically in the range of 10^9^ to 10^10^ dm^3^ mol^–1^ s^–1^.^14,15^

For modeling homogeneous nucleation from solution, the solute species A and B are replaced by the solvated monomeric solute molecules S_*x*_M (the symbol S_*x*_M indicates that each solute molecule M is solvated by *x* solvent molecules S) and *k*_2_ is replaced by the nucleation rate constant k*. Diffusion-driven collisions of monomeric solvated solute molecules create a steady-state population of precursors ${\left(S_{x}M\right)}_{n}$ containing *n* S_*x*_M complexes, up to a maximum size of *n* = *n*_max_

13 $$S_{x}M\begin{array}{l} \overset{\begin{array}{l} +S_{x}M\\ k_{1} \end{array}}{\to }\\ \underset{\begin{array}{l} k_{-1}\\ -S_{x}M \end{array}}{\leftarrow } \end{array}{\left(S_{x}M\right)}_{2}\begin{array}{l} \overset{\begin{array}{l} +S_{x}M\\ k_{1} \end{array}}{\to }\\ \underset{\begin{array}{l} k_{-1}\\ -S_{x}M \end{array}}{\leftarrow } \end{array}{\left(S_{x}M\right)}_{3}\begin{array}{l} \overset{\begin{array}{l} +S_{x}M\\ k_{1} \end{array}}{\to }\\ \underset{\begin{array}{l} k_{-1}\\ -S_{x}M \end{array}}{\leftarrow } \end{array}...\begin{array}{l} \overset{\begin{array}{l} +S_{x}M\\ k_{1} \end{array}}{\to }\\ \underset{\begin{array}{l} k_{-1}\\ -S_{x}M \end{array}}{\leftarrow } \end{array}{\left(S_{x}M\right)}_{n_{\max}}$$

As shown in the Supporting Information (Section S1), the size distribution of these precursors is determined by the nominal solute concentration [S_*x*_M]_0_ of the overall solution (in units of mol dm^–3^) according to

14 $$\left[{\left(S_{x}M\right)}_{n}\right]=\frac{1}{K}{\left(\frac{\sqrt{4{K\left[S_{x}M\right]}_{0}+1}-1}{\sqrt{4{K\left[S_{x}M\right]}_{0}+1}+1}\right)}^{n}$$

where *K* is the equilibrium constant defined in eq 10 and [(S_*x*_M)_*n*_] is the steady-state concentration (again, in mol dm^–3^) of the precursors containing *n* solvated solute molecules. This equation has previously been derived also in the context of analysing solute aggregation effects in phthalocyanine solutions.^73^

In line with modeling of the solution as an ideal solution of weakly interacting solvated solute molecules, the diffusion-driven collisions of solvated solute monomers create spatial proximity of the solvated solute monomers in the precursors, but their solvation shell remains intact. Indeed, local structure probes such as near-edge X-ray absorption fine-structure (NEXAFS) and pair-distribution function analysis have indicated that the average solvation shell around imidazole molecules in aqueous solution remains unchanged even at extremely high solute concentrations.^17–19^ In stable solutions, the formation of the precursors is therefore reversible, i.e., diffusion of the constituent solvated solute monomers also leads to their dissociation on time scales of nanoseconds, as determined by eq 12. The precursors (S_*x*_M)_*n*_ can thus be viewed as diffusion-driven local solute density fluctuations (SDFs) in the solution.

The assumption that a real solution can be considered an ideal solution of solvated solute molecules implies that interactions between solvated solute molecules are similar in strength to solvent–solvated solute and solvent–solvent interactions. Residual attractive interactions, for example due to polarization of solute molecules in the solvation shell, are weaker than thermal energies driving diffusion, i.e., on the order of less than a few *k*_B_ *T*. The rates of monomer attachment and detachment to the precursors should then be approximately equal: *k*_1_ ≈ *k*_–1_. Consequently, the pre-equilibrium constant *K* also becomes *K* ≈ 1, as required by eq 10, and the free energy change in the overall solution associated with local solute density fluctuations is Δ*G* = −*RT* ln *K* ≈ 0. Local property variations in the regions of high density, most importantly the associated local chemical potential increase, are compensated by opposing property variations in the solution surroundings. The assumption *K* = 1 was the basis for the previously reported analysis of homogeneous nucleation rates in solution.^12^

Equation 14 predicts that a saturated solution contains regions of high solute density to a maximum size of n* solute molecules. As shown in the Supporting Information (Section S2), n* can be predicted when [S_*x*_M]_0_ is replaced with the saturation concentration [S_*x*_M]_0_^*^

15 $$n^{*}=\frac{\ln\left(K\right)-\ln\left(N_{A}V\right)}{\ln\left(\frac{\sqrt{4K{\left[S_{x}M\right]}_{0}^{*}+1}-1}{\sqrt{4K{\left[S_{x}M\right]}_{0}^{*}+1}+1}\right)}$$

where *V* is the volume of the solution and *N*_A_ Avogadro’s number.

Nucleation rates are commonly reported as a function of the supersaturation ratio *S*, which follows from the difference between the chemical potential of the solutes in the supersaturated solution with activity *a*_0_

16 $$\mu_{0}=\mu_{0}^{⊖}+RT\;\ln\left(a_{0}\right)$$

and the chemical potential of the solutes in the saturated solution (having the activity *a*_0_^*^)­

17 $$\mu_{0}^{*}=\mu_{0}^{⊖}+RT\;\ln\left(a_{0}^{*}\right)$$

through

18 $$\Delta \mu =\mu_{0}-\mu_{0}^{*}=RT\;\ln\left(\frac{a_{0}}{a_{0}^{*}}\right)\equiv RT\;\ln S$$

where *R* is the universal gas constant and μ_0_^⊖^ the standard chemical potential. Δμ is referred to as the supersaturation.^7^ For an ideal solution, the activities can be replaced by the concentrations, resulting in the relationship

19 $${\left[S_{x}M\right]}_{0}=S{\left[S_{x}M\right]}_{0}^{*}$$

## 2 Rate Equation for Nucleation from Large Solute Density Fluctuations (LSDFs)

For supersaturated solutions (*S* > 1), eq 14 predicts the formation of an excess of large solute density fluctuations (LSDFs), defined as regions of high solute density containing at least n* solute molecules, i.e., having a size larger than or equal to the largest SDF in a saturated solution. The concentration of LSDFs can be calculated from eq 14 by reference to the saturation concentration of the solution, [S_*x*_M]_0_^*^, according to

20 $$\left[LSDF\right]=\sum_{n=n^{*}}^{\infty }\left(\left[{\left(S_{x}M\right)}_{n}\right]-{\left[{\left(S_{x}M\right)}_{n}\right]}^{*}\right)$$

where [(S_*x*_M)_*n*_]^*^ is the concentration of dense regions with *n* solute molecules in the saturated solution (i.e., for *S* = 1).

The absolute number of LSDFs in a solution volume *V* is given by *N*_A_ *V*[LSDF]. For *K* = 1, this quantity has previously been shown to be proportional to experimentally observed supersaturation-dependent homogeneous nucleation rates for 11 different solute–solvent combinations.^12^ The proportionality suggests that LSDFs are indeed the loci of homogeneous nucleation events. The extremely high supersaturation in the LSDFs appears to set up the spatiotemporal molecular level conditions required for the spontaneous formation of crystal nuclei. The proportionality between the number of LSDFs and the observed nucleation rates also implies that the transformation of LSDFs into nuclei is a first order rate process. The first order rate constant k* was identified as the nucleation rate constant, which is system-specific and independent of the overall solute concentration (and hence also of the supersaturation of the solution). This independence is expected because the local solute density within the LSDFs, and hence the physical properties within an LSDF do not depend strongly on their size *n*.

Using eq 14 we can examine how non-zero interactions between solvated solutes in a non-ideal solution may influence the quantity [LSDF], by adjusting the value of *K* to include attractive (*K* > 1) or repulsive (0 < *K* < 1) interactions. Generally, attractive interactions between solvated solutes extend the size distribution of the SDFs toward larger sizes at the expense of the concentration of smaller SDFs and solvated solute monomers. This is perhaps most easily seen in the expression for n* in eq 15: for a given solution volume *V*, the term ln­(*K*) in the numerator increases with *K* while ln­(*N*_A_ *V*) is constant and the numerator only weakly dependent on *K*. The corresponding effect on the monomer population is illustrated in Section S1 of the Supporting Information.

Light scattering techniques have provided evidence for mesoscale clusters of organic solutes in solution, with sizes on the order of several 10s to 100s of nm.^20–22^ Their presence has been invoked in discussions of nonclassical nucleation pathways, where it has been suggested that these mesoscale clusters are prenucleation phases.^20–22^ The formation of such mesoscale phases has also been associated with observations of homogeneous nucleation rate variations due to thermal pretreatments and aging of solutions.^21,23^ Their origin, molecular nature and stability are at this point of time still unclear^20^ and require more research. They are mentioned here because it may seem tempting to consider the largest SDFs as candidates for such mesoscale clusters, but even in solutions favoring the formation of large SDFs (high nominal solute concentrations, strong attractive solute–solute interactions) their size does not exceed 100s of solvated solute molecules, which is insufficient to be compatible with mesoscale solute aggregates on the order of 10s and 100s of nm. For example, a calculation of n* for 20 cm^3^ of solution volume with a nominal solute concentration of 1 mol dm^–3^ and assuming attractive solute–solute interactions of 3 *k*_B_ *T* yields a value of 214, which is orders of magnitude smaller than required for the reported^20–22^ mesoscale cluster sizes on the order of ∼100 nm. The formation of observable, stable mesophases would suggest an irreversible process of forming a distinct dense phase, which would have to be associated with values of *K* corresponding to solvated solute interactions stronger than a few *k*_B_ *T*. Diffusion-driven SDFs are not such metastable phases, and should therefore not be confused with metastable phases or prenucleation phases considered in contemporary discussions of nonclassical nucleation pathways.^4,9,20,24–34^ This is not to say that metastable mesophases or prenucleation clusters generally do not exist. They may well have a role in nucleation as intermediate phases, and it may even be possible to model their influence based on eq 14, for example by assuming sufficiently large values of *K* to model attractive interactions driving their formation. Such an analysis has previously been carried out for phthalocyanine aggregation in solution.^73^ Other possible mechanisms, for example seeding of mesoscale clusters by nanoscale impurities or incomplete/partial dissolution of solute crystals in the preparation of solutions, may also be at work. As has been discussed in some depth before,^12^ heterogeneous impurities in solutions are extremely difficult to avoid, and their nature and role in homogeneous nucleation from solution needs more systematic research.

For extremely high supersaturation ratios (far exceeding the values of *S* covered in the analysis in this paper), liquid–liquid phase separations (LLPSs) may also take place. Indeed, for high values of *K* extremely large SDFs with lifetimes permitting their direct observation may exist. The stabilization of solute molecules in a dense liquid phase formed by an LLPS may for example explain decreasing nucleation rates at very high supersaturations, which have been on the scientific record for a long time.^2^ It would be interesting to examine such mechanistic scenarios more systematically with the kinetics framework developed in this paper, but they are beyond its scope.

In the Supporting Information Section S3, the effects of varying *K* and the supersaturation ratio *S* on the concentration of LSDFs relative to ideal solution (*K* = 1) are plotted for volumes of 1 cm^3^ (relevant for the l-histidine analysis in this paper) and for 20 cm^3^ (relevant for the salicylic acid data analyzed in this paper). The plots cover solutions with nominal bulk concentrations from 0.01 mol dm^–3^ to 5 mol dm^–3^ and supersaturation ratios up to 6. In practice, the most relevant concentrations are ∼0.05 to ∼0.5 mol dm^–3^ and typical supersaturation ratios are in the range 1.1 to 2.2. For these, the influence of *K* turns out to be weak, exceeding a factor of 10 only for some exceptional combinations of very low nominal solute concentrations (≲0.05 mol dm^–3^) and strong attraction between solvated solutes. Moderately attractive and repulsive interactions (associated with an energy of 1 *k*_B_ *T*) are for all systems reported in this and a previous paper^12^ associated with variations of less than 10.

The main reason for the weak influence of *K* on the nucleation rate is that eq 20 describes in effect a supersaturation-induced perturbation of the population of SDFs in the saturated system. Solution nonideality (*K* ≠ 1) influences both the concentrations [(S_*x*_M)_*n*_]^*^ and [(S_*x*_M)_*n*_] of the SDFs in the reference saturated solution and the supersaturated solution, respectively. The absolute concentrations [(S_*x*_M)_*n*_] and [(S_*x*_M)_*n*_]^*^ vary strongly as a function of *K*, but their difference varies weakly. In practice, the value of *K* is not known and its effect is difficult to separate from k* as it is comparatively small and just an approximately constant correction factor. It is therefore more practical to determine and report the nucleation rate constants k* assuming *K* = 1. Where desired and possible, the influence of attractive and repulsive solute–solute interactions can then be considered later and introduced as a correction factor. For currently available nucleation rate data the magnitude of such corrections will often be below the accuracy and reproducibility of the experimentally determined values.

It is interesting that the plots in Section S3 make predictions that may be tested by more accurate future nucleation rate measurements. For example, attractive solute–solute interactions are in principle expected to increase homogeneous nucleation rates through an increase in the concentration of LSDFs, while repulsive interactions decrease the rates. For some low supersaturations and low nominal solution concentrations the reverse is predicted because absolute SDF concentrations are also influenced by the mass balance condition. This comes into play for example when attractive solute–solute interactions shift the SDF size distribution to larger sizes. The SDFs then remove, on average, more solute monomers from the solution but are fewer in number, reducing the absolute number of LSDFs in solutions with low supersaturation. It should also be noted that the counting of LSDFs with integer sizes according to eq 20 can introduce large rounding errors in the predicted supersaturation-dependence of the rates when the LSDFs are very small. For n* values below 10 care should be exercised for repulsive interactions exceeding 1 *k*_B_ *T* and/or very low nominal solution concentrations (≲0.05 mol dm^–3^).

Equation 20 is easily extended to formulate a new equation for the homogeneous nucleation rate J* of single component crystals from solution^12^

21 $$J^{*}=k^{*}N_{A}\sum_{n=n^{*}}^{\infty }\left(\left[{\left(S_{x}M\right)}_{n}\right]-{\left[{\left(S_{x}M\right)}_{n}\right]}^{*}\right)=k^{*}N_{A}\left[LSDF\right]$$

Equation 21 is the equivalent of the reaction-limited bimolecular rates in eqs 6 and 9. It is important to recall that eq 21 is obtained assuming that *k*_–1_ ≫ k*, with *k*_–1_ having typical values of 10^9^ to 10^10^ dm^3^ mol^–1^ s^–1^ (vide supra). Previously reported experimentally determined nucleation rate constants k* had values of 10^–3^ s^–1^ and lower,^12^ validating the assumption of *k*_–1_ ≫ k*. They are therefore also in line with the hypothesis that the rate-determining step is nucleation from LSDFs with the first order rate constant k*. Equation 21 has the mathematical form of a first order rate equation describing unimolecular rate processes^14,35^ according to

22 $${\left(S_{x}M\right)}_{n}\overset{\begin{array}{l} -mxS\\ -\left(n-m\right)S_{x}M\\ k^{*} \end{array}}{\to }M_{m}$$

with *n* ≥ n*. The formation of a crystal nucleus containing *m* molecules is associated with desolvation events that release *mx* solvent molecules and (*n* – *m*) solvated solute molecules S_*x*_M. The likelihood that the resulting nucleus M_*m*_ continues to grow to a macroscopically detectable crystal depends on the concentration [S_*x*_M]_0_ of solvated solute monomers S_*x*_M in the solution as well as the rate constants *k*_att_ and *k*_det_ for attachment of solvated solute monomers to the nucleus and detachment from it, respectively. For *S* > 1 the nucleus M_*m*_ will continue to grow to a macroscopic crystal M_∞_, while for *S* < 1 it will redissolve into the solution, reforming m S_*x*_M molecules

23 $$S_{x}M\begin{array}{l} \overset{\begin{array}{l} +S_{x}M\\ \begin{array}{l} -xS\\ k_{att} \end{array} \end{array}}{\to }\\ \underset{\begin{array}{l} k_{\det}\\ +xS \end{array}}{\leftarrow } \end{array}M_{2}\begin{array}{l} \overset{\begin{array}{l} +S_{x}M\\ \begin{array}{l} -xS\\ k_{att} \end{array} \end{array}}{\to }\\ \underset{\begin{array}{l} k_{\det}\\ +xS\\ -S_{x}M \end{array}}{\leftarrow } \end{array}...\begin{array}{l} \overset{\begin{array}{l} +S_{x}M\\ \begin{array}{l} -xS\\ k_{att} \end{array} \end{array}}{\to }\\ \underset{\begin{array}{l} k_{\det}\\ +xS\\ -S_{x}M \end{array}}{\leftarrow } \end{array}M_{m-1}\begin{array}{l} \overset{\begin{array}{l} +S_{x}M\\ \begin{array}{l} -xS\\ k_{att} \end{array} \end{array}}{\to }\\ \underset{\begin{array}{l} k_{\det}\\ +xS\\ -S_{x}M \end{array}}{\leftarrow } \end{array}M_{m}\begin{array}{l} \overset{\begin{array}{l} +S_{x}M\\ \begin{array}{l} -xS\\ k_{att} \end{array} \end{array}}{\to }\\ \underset{\begin{array}{l} k_{\det}\\ +xS\\ -S_{x}M \end{array}}{\leftarrow } \end{array}M_{m+1}\begin{array}{l} \overset{\begin{array}{l} +S_{x}M\\ \begin{array}{l} -xS\\ k_{att} \end{array} \end{array}}{\to }\\ \underset{\begin{array}{l} k_{\det}\\ +xS\\ -S_{x}M \end{array}}{\leftarrow } \end{array}...\begin{array}{l} \overset{\begin{array}{l} +S_{x}M\\ \begin{array}{l} -xS\\ k_{att} \end{array} \end{array}}{\to }\\ \underset{\begin{array}{l} k_{\det}\\ +xS\\ -S_{x}M \end{array}}{\leftarrow } \end{array}M_{\infty }$$

This system of coupled equilibria provides a framework for integrating homogeneous nucleation rates with an analysis of crystal growth rates.

## 3 Temperature Dependence: Eyring–Polanyi Rate Equation

According to absolute rate theory,^14,35,36^ the rate constant k* should exhibit an Arrhenius-dependence on temperature and allow a nucleation rate analysis within the framework of absolute rate and transition state theory.^15,36–39^ Combining eq 21 with the Eyring–Polanyi expression^14,35–37^ for the temperature-dependence of the rate constant should predict the temperature-dependence of homogeneous nucleation rates.

The rate constant k* can be expressed as^35,36^

24 $$k^{*}=\kappa \frac{k_{B}T}{h}\;\exp\left(-\frac{{\Delta G}^{‡}}{RT}\right)$$

This equation contains the activation energy Δ*G* ^⧧^, which is the free energy change associated with the formation of the activated molecular complex in the transition state to the formation of crystal nuclei M_*m*_ from solvated solute molecules. The reported proportionality between the number of LSDFs and experimental nucleation rates^12^ suggests that the activated complex is located inside an LSDF with a size *n* > n*. The activated complex represents a collection of solute and solvent molecules in a metastable state that exhibits the spatiotemporal alignment required to facilitate the formation of a crystal nucleus. More formally put, it is the molecular transition state located at a saddle point on the potential surface comprising all possible spatiotemporal configurations of solute and solvent molecules in the LSDF and in the product suspension containing the nucleus M_*m*_ and the released solvent molecules. In eq 24, κ is a transmission coefficient that accounts for the fact that not every activated complex crossing the activation barrier will lead to a nucleation event. κ is unknown but expected to be close to 1 and will therefore be taken as such from now on.

With Δ*G* ^⧧^ = Δ*H* ^⧧^ – *T*Δ*S* ^⧧^, we can introduce the activation enthalpy Δ*H* ^⧧^ and activation entropy Δ*S* ^⧧^ associated with forming the transition state, giving the classic Eyring–Polanyi expression^14,36,37,41–43^ for the rate constant

25 $$k^{*}=\kappa \frac{k_{B}T}{h}\;\exp\left(\frac{{\Delta S}^{‡}}{R}\right)\;\exp\left(-\frac{{\Delta H}^{‡}}{RT}\right)$$

The values of Δ*H* ^⧧^ and Δ*S* ^⧧^ can be determined from temperature-dependent nucleation rate measurements^36^ (vide infra). They are, respectively, the activation enthalpy and activation entropy associated with the formation of the activated complex. Combining with eq 21, we obtain an Eyring–Polanyi rate equation for homogeneous nucleation from solution

26 $$J^{*}=\kappa \frac{k_{B}T}{h}\;\exp\left(\frac{{\Delta S}^{‡}}{R}\right)\left[LSDF\right]N_{A}\;\exp\left(-\frac{{\Delta H}^{‡}}{RT}\right)$$

Superficially, there are similarities between this expression and the CNT rate eq 2. Both contain a pre-exponential factor with a number density of nucleation centers and an exponential activation term. However, there is a fundamental difference in that the supersaturation ratio *S* influences the absolute rate J* only through the concentration of LSDFs, which is determined by the overall concentration [S_*x*_M]_0_ of the solution and hence *S* via eqs 14 and 19. The resulting number density of LSDFs in the solution volume *V*, *N*_A_[LSDF], is a minuscule quantity relative to the corresponding number density $C_{0}{=N_{A}\left[S_{x}M\right]}_{0}$ in the CNT eq 2. However, the concentration [LSDF] increases quasi-exponentially with the supersaturation ratio *S* (see Figure 1 and the associated discussion below) and is thus responsible for the characteristic quasi-exponential increase of the nucleation rate with the supersaturation ratio.^12^ The remaining terms in J* are independent of *S*, reflecting the fact that the physical properties within an LSDF do not depend strongly on the overall concentration of the solution. This contrasts with CNT, where the supersaturation ratio *S* is responsible for the quasi-exponential increase of supersaturation-dependent nucleation rates through its inclusion in the exponential activation term, as expressed through eq 2, and only somewhat through the pre-exponential mass transport terms.

**Figure 1:**
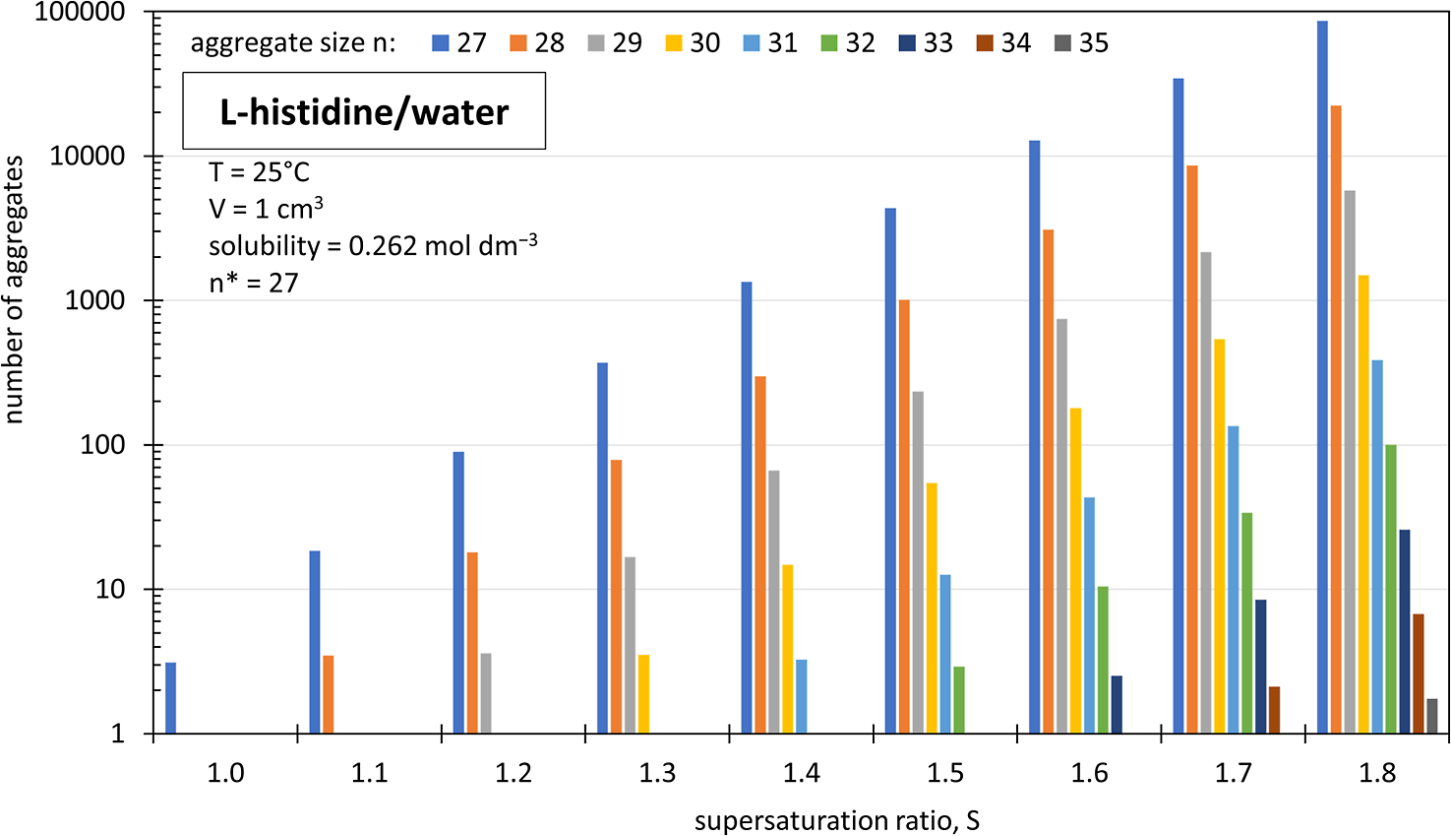
Predicted supersaturation-dependent size distribution of LSDFs with *n* ≥ n* = 27 of solvated l-histidine molecules in 1 mL aqueous solution (calculated as *N*_A_ *V*[LSDF]). Note the logarithmic scale on the ordinate.

This Eyring–Polanyi formulation of the nucleation rate identifies characteristic enthalpic and entropic activation parameters (Δ*H* ^⧧^, Δ*S* ^⧧^) that are readily determined by temperature-dependent rate measurements. Rearranging eq 25 predicts that ln­(k*/*T*) should decrease linearly with the inverse of the absolute temperature, providing values for Δ*H* ^⧧^ and Δ*S* ^⧧^ from the slope and intercept of the resulting plot, respectively, through

27 $$\ln k^{*}-\ln\left(\frac{k_{B}T}{h}\right)=\frac{{\Delta S}^{‡}}{R}-\frac{{\Delta H}^{‡}}{RT}$$

One can thereby predict homogeneous nucleation rates for all supersaturations and temperatures with only two a priori unknown system-specific constants: the activation enthalpy Δ*H* ^⧧^ and the activation entropy Δ*S* ^⧧^. In principle, these could be determined from two accurate nucleation rate measurements at two different temperatures.

## 4 Results

### 4.1 Homogeneous Nucleation of l-Histidine from Aqueous Solution

Homogeneous nucleation rates of l-histidine in water are well documented^11,44^ and have previously been used in the context of demonstrating practical nucleation rate analysis.^11^ The solubility of l-histidine in water at a temperature of 25 °C was reported to be 0.262 mol dm^–3^, alongside experimental homogeneous nucleation rates for supersaturation ratios *S* of 1.55, 1.60, 1.64, 1.69 and 1.74, in a solution volume of 1 cm^3^ (Table 1).^11^ Equation 15 predicts that the largest LSDF in a volume of 1 cm^3^ saturated aqueous l-histidine contains n* = 27 solvated l-histidine molecules. Figure 1 indicates the supersaturation-dependent size distributions of LSDFs per cm^3^ of solution, as calculated with eq 14. As the supersaturation ratio is increased to 1.8, LSDFs with sizes up to *n* = 35 appear. With eq 20, the summation of aggregates with *n* ≥ n* for each supersaturation ratio gives *N*_A_ *V*[LSDF], the number of excess LSDFs. Explicit supersaturation-dependent *N*_A_ *V*[LSDF] values as a function of supersaturation are given in Table 1.

**Table 1:** Summary of Experimental^11^ (*J*) and Calculated (J*) Homogeneous Nucleation Rates for the l-Histidine/Water System at *T* = 25 °C

*S*	[S_*x*_M]_0_/mol dm^–3^	*V*/cm^3^	*N*_A_ *V*[LSDF]	*J*/s^–1^ m^–3^	J*/s^–1^ m^–3^	k*/s^–1^
1.00	0.26	1	0		0	3.3 × 10^–8^
1.25	0.33	1	231		8	3.3 × 10^–8^
1.35	0.35	1	914		30	3.3 × 10^–8^
1.45	0.38	1	3174		105	3.3 × 10^–8^
1.55	0.41	1	9890	160	326	3.3 × 10^–8^
1.60	0.42	1	16,850	630	556	3.3 × 10^–8^
1.64	0.43	1	25,412	690	839	3.3 × 10^–8^
1.69	0.44	1	41,709	1870	1376	3.3 × 10^–8^
1.74	0.46	1	67,179	1980	2217	3.3 × 10^–8^
1.85	0.48	1	180,425		5954	3.3 × 10^–8^

Multiplying the calculated *N*_A_ *V*[LSDF] values with a rate constant k* of 3.3 × 10^–8^ s^–1^ and dividing by the solution volume achieves excellent agreement between calculated and experimental^11^ nucleation rates (Table 1, Figure 2). All calculated parameters and rates are shown in Table 1 alongside the experimental data.

**Figure 2:**
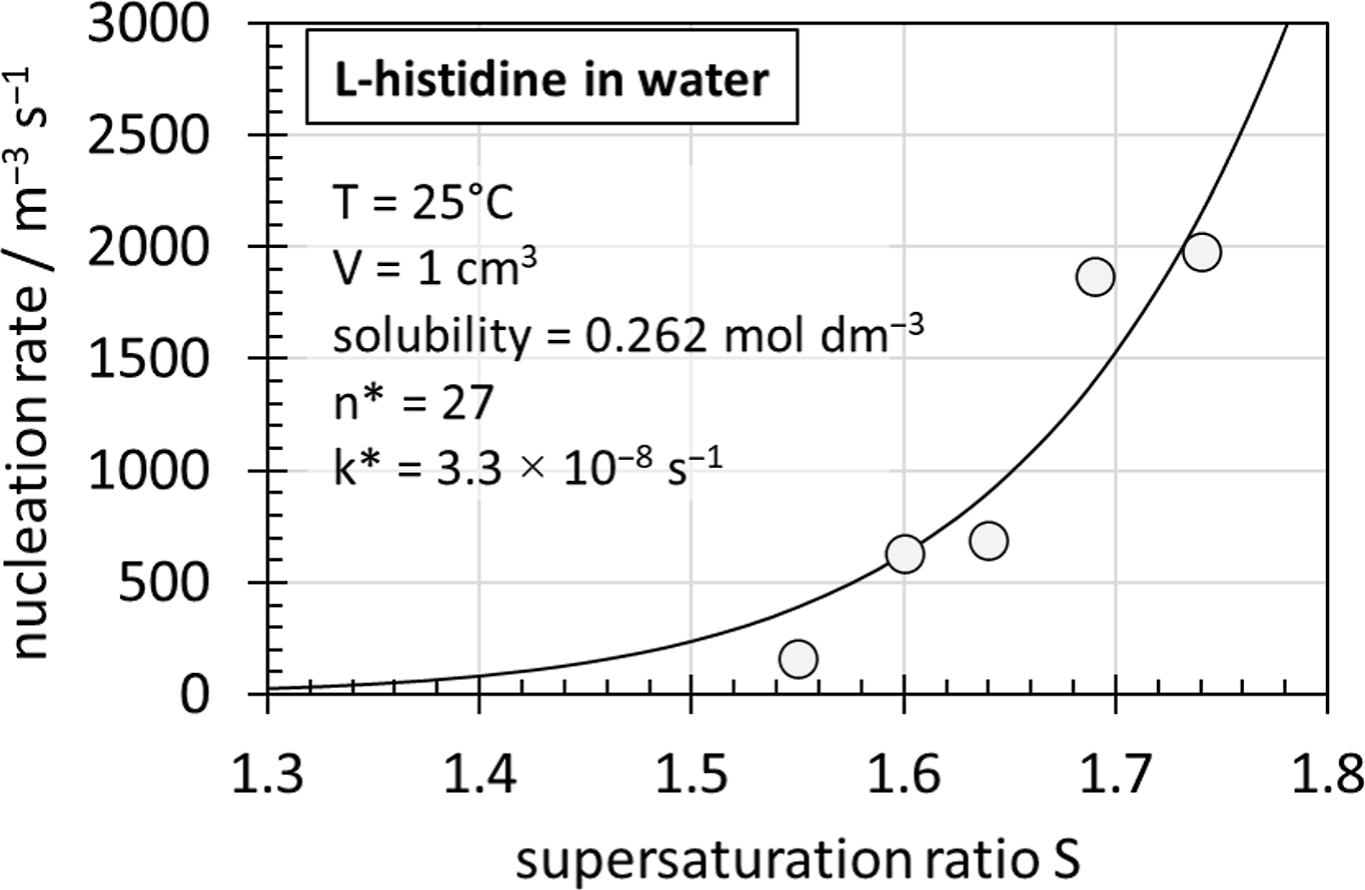
Nucleation rates J* (line) determined with eq 21 by a least-squares best fit against experimental^11^ *J* data.

Temperature-dependent nucleation rates of l-histidine in water^44^ were analyzed with eq 26. As for the example above, k* values were determined by least-squares fitting against the experimental^44^ nucleation rate data. The calculated temperature-dependent nucleation rates (Figure 3) reproduce all trends in the experimental data very well. The associated calculated parameters, including the determined values of k*, are assembled in Table 2. The rate of homogeneous nucleation increases with temperature, as one would expect for an activated process. This is easily seen in Table 2 when noting that the overall concentrations [S_*x*_M]_0_ at the four different temperatures are comparable, spanning values from approximately 0.42 to 0.52 mol dm^–3^. The higher the temperature, the less supersaturation is required to achieve comparable nucleation rates. Accordingly, the absolute rate analysis with J* finds that the required number *N*_A_ *V*[LSDF] of excess LSDFs decreases with temperature, while the rate constant k* increases.

**Figure 3:**
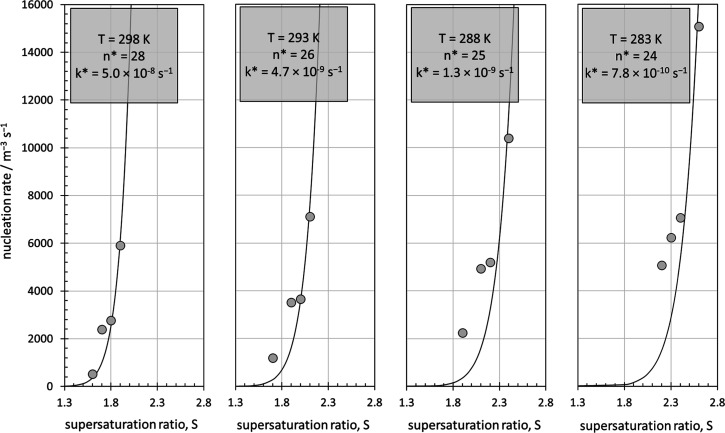
Temperature-dependent experimental^44^ nucleation rates (data points) vs calculated nucleation rates J* (line) with rate constants k* determined by a least-squares best fit with eq 21.

**Table 2:** Experimental^44^ and Calculated Rates for the Temperature-Dependent Nucleation of Aqueous l-Histidine

*T*/K	*S*	[S_*x*_M]_0_/mol dm^–3^	*V*/cm^3^	*J*/s^–1^ m^–3^	*N*_A_ *V*[LSDF]	J*/s^–1^ m^–3^	k*/s^–1^
283.15	2.2	0.43	1	5063	1,928,986	1504	7.8 × 10^–10^
	2.3	0.45	1	6233	3,694,649	2882	
	2.4	0.47	1	7062	6,826,100	5324	
	2.6	0.51	1	15,070	21,180,592	16,521	
288.15	1.9	0.42	1	2240	270,152	349	1.3 × 10^–9^
	2.1	0.46	1	4940	1,244,481	1605	
	2.2	0.48	1	5207	2,490,941	3213	
	2.4	0.53	1	10,401	8,874,882	11,449	
293.15	1.7	0.42	1	1185	59,804	279	4.7 × 10^–9^
	1.9	0.46	1	3506	349,141	1630	
	2.0	0.49	1	3658	771,783	3604	
	2.1	0.51	1	7112	1,621,714	7573	
298.15	1.6	0.43	1	517	7056	353	5.0 × 10^–8^
	1.7	0.46	1	2382	19,756	988	
	1.8	0.49	1	2773	51,229	2561	
	1.9	0.51	1	5914	124,176	6209	

The rate constant k* is extremely temperature-dependent, especially when compared to typical values for chemical reactions.^36^ Varying the temperature by 15 K changes the rate constant by almost 2 orders of magnitude (Table 2). Inspection of the literature confirms that homogeneous organic crystal nucleation rates commonly exhibit strong temperature dependencies, for example in the cases of *p*-aminobenzoic and salicylic acids.^45–47^ Such a strong temperature dependence of a rate constant indicates a high activation barrier. Indeed, an Eyring–Polanyi plot according to eq 27 yields values of Δ*H* ^⧧^ = 190 ± 40 kJ mol^–1^ and Δ*S* ^⧧^ = 250 ± 150 J K^–1^ mol^–1^ (Figure 4). The error margins stem from the scatter in the limited data set of nucleation rate data available, so absolute values should not be overinterpreted. The values for Δ*H* ^⧧^ and Δ*S* ^⧧^ indicate that Δ*G* ^⧧^ is approximately 115 kJ mol^–1^ in the temperature range of the experimental nucleation rate data.

**Figure 4:**
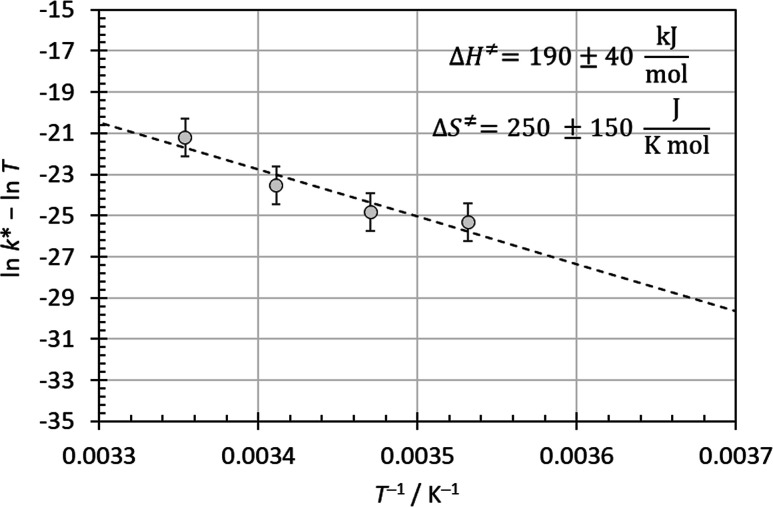
Eyring–Polanyi plot for temperature-dependent homogeneous nucleation rates of l-histidine in aqueous solution according to eq 27. κ was assumed to be equal to 1.

The high values for Δ*S* ^⧧^ and Δ*H* ^⧧^ suggest that the formation of the activated complex for homogeneous crystal nucleation requires multiple, possibly concerted, solute aggregation and desolvation events. An activation barrier associated with multiple solute–solute and solute–solvent bond-forming and bond-breaking events is well-known from other branches of chemistry, e.g., heterogeneous catalysis.^48^ The high activation entropy appears to be an example of a multiexcitation entropy (MEE).^48,49^

l-Histidine has been reported to have a hydration sphere containing approximately 9 water molecules at a temperature of 283.15 K, decreasing to almost 4 when the temperature is raised to 313.15 K.^50^ The most strongly bound water molecules in the hydration shell are expected to be hydrogen-bonded to l-histidine, with bond strengths on the order of 10–20 kJ mol^–1^.^51^ The determined activation enthalpy of ∼190 kJ mol^–1^ thus suggests that the formation of the activated complex may involve breaking of 10–20 solute–solvent bonds, not accounting for any enthalpic offset due to the formation of solute–solute bonds in the transition state. This estimate for the size of the activated complex is compatible with the predicted size of the LSDFs, which contain significantly more (≥27) hydrated l-histidine molecules. Moreover, using the previously published solubility data^44^ an enthalpy of solution of 15.2 kJ mol^–1^ can be determined. The activation enthalpy for nucleation of ∼190 kJ mol^–1^ is approximately 12.5 times this value, which suggests that overcoming the activation barrier for the formation of a crystal nucleus (the reverse process of crystal dissolution) requires the separation of approximately a dozen solute molecules from the solution. This point will be revisited in the discussion below.

### 4.2 Solvent-Dependent Homogeneous Nucleation of Salicylic Acid

Temperature-dependent nucleation rates at different supersaturation ratios have been reported for salicylic acid (SA) in 6 different solvents.^46,47^ These studies were performed by preparing a single saturated solution of SA in each solvent at elevated temperature, and performing induction time measurements from supersaturated solutions at lower temperatures. It was found that the formation of salicylic acid crystals becomes increasingly difficult (requiring higher supersaturation ratios) in the order

chloroform<ethylacetate<acetonitrile<acetone<methanol<aceticacid

Within the framework of CNT, higher supersaturation ratios are associated with higher nucleation works *RT* ln *S*. Crystal formation requiring a higher supersaturation ratio is therefore considered more “difficult” than in a system requiring less supersaturation. Raman spectroscopy studies of solute–solvent binding in the solutions were combined with a determination of enthalpies of solution and a density functional theory (DFT) analysis of solvent–solute and solvent–solvent interactions.^46^ This analysis indicated that the strength of solute–solvent interactions and hence the desolvation rate was the decisive factor determining the activation barrier to crystal nucleation in each solvent.

The Eyring–Polanyi analysis of the reported experimental nucleation rate data^47^ could be performed for solutions in the four solvents ethyl acetate, acetonitrile, methanol and acetic acid (Figure 5, Table 3). The dependence of the nucleation rate constants on the inverse temperature was found to be linear, confirming an Arrhenius temperature dependence of the homogeneous nucleation rate. As observed previously,^12^ for a given supersaturation, the increasing values of the nucleation rate constant k* are associated with increasing nucleation rates. The values of k* are highest for nucleation from ethyl acetate and lowest for acetic acid solutions (see also Table 3). For inverse temperatures below approximately 0.00328 K^–1^ (i.e., above ∼305 K) nucleation rates from acetonitrile rank second after ethyl acetate, while nucleation from methanol ranks third. The Eyring–Polanyi plot thus expresses the previously reported^46,47^ order of the ease of salicylic acid nucleation in the four solvents

ethylacetate>acetonitrile>methanol>aceticacid

but it also provides additional insight. First, it reveals that at temperatures below ∼305 K the nucleation rate constants of methanol and acetonitrile switch their numerical order; nucleation from methanol is then associated with a lower activation barrier than for nucleation from acetonitrile. Second, it reveals the reason for this switch: both the slope and the *y*-intercept of the Eyring–Polanyi plot for methanol are lower than for acetonitrile, revealing (Table 3) that the activation enthalpy (determined from the slope) and entropy (determined from the *y*-intercept) for nucleation from methanol are lower than for acetonitrile. The difference between “easy” and “hard” nucleation is not only determined by the strength of solvent–solute interactions^46^ but turns out to be additionally influenced by the entropic contribution to the free energy of activation. The relationship between activation enthalpy and activation entropy will be examined in more detail in the discussion section below.

**Figure 5:**
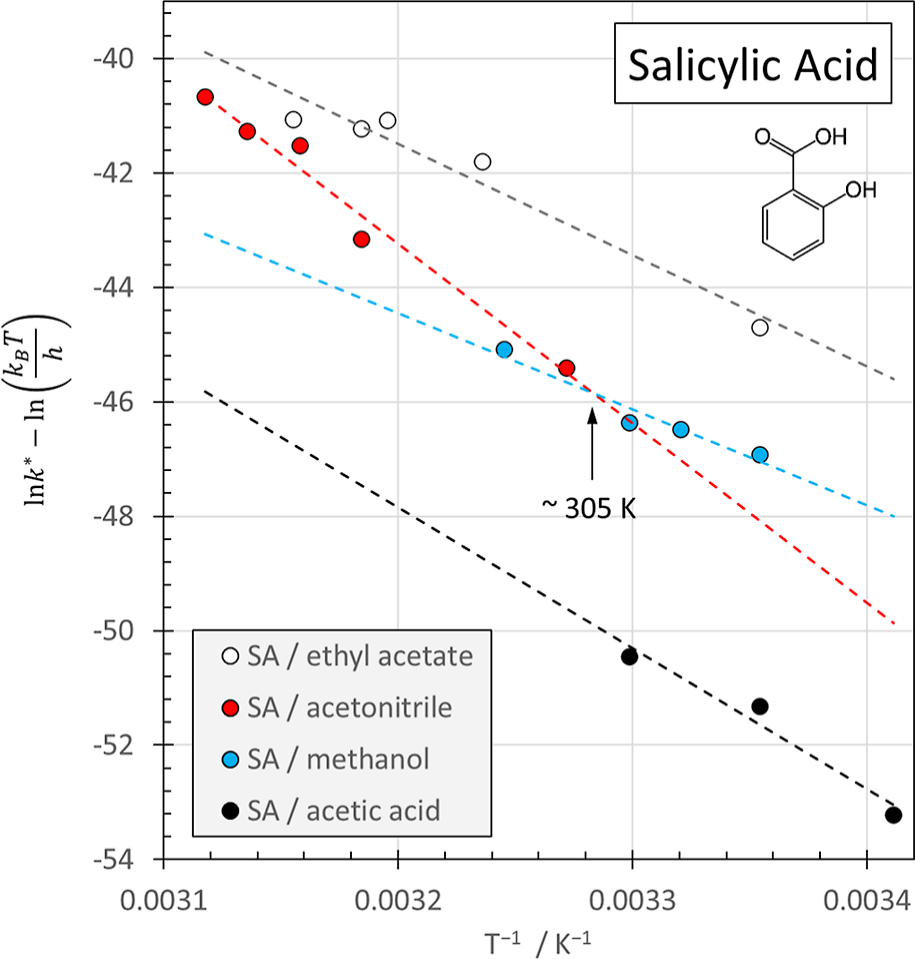
Eyring–Polanyi plots for the temperature-dependent homogeneous nucleation rates^47^ of salicylic acid in ethyl acetate, acetonitrile, methanol and acetic acid according to eq 27. κ was assumed to be equal to 1. The crossover temperature at ∼305 K for the nucleation rate constants of methanol and acetonitrile is indicated in the figure.

**Table 3:** Temperatures *T*, Solubilities [S_*x*_M]_0_^*^,^47^ Experimental Nucleation Rates *J*,^47^ Calculated Maximum LSDF Size n* at [S_*x*_M]_0_^*^, Nucleation Rate Constants k*, Activation Enthalpies Δ*H* ^⧧^, Activation Entropies Δ*S* ^⧧^, Enthalpies of Solution Δ*H*_solution_,^47^ and Solvent–Solvent Binding Energies per mol of Solvent^47^ for Salicylic Acid Crystallization from Ethyl Acetate, Acetonitrile, Methanol and Acetic Acid

	*T*/K	${\left[S_{x}M\right]}_{0}^{*}/mol\;{dm}^{-3}$	*J*/m^–3^ s^–1^	n*	k*/s^–1^	Δ*H* ^⧧^/kJ mol^–1^	Δ*S* ^⧧^/J K^–1^ mol^–1^	Δ*H*_solution_/kJ mol^–1^
ethyl acetate	316.95	1.81	13	69	1.52 × 10^–5^	161.8	172.8	13.06
	314.05	1.74	28	68	8.55 × 10^–6^			
	312.95	1.72	35	68	6.85 × 10^–6^			
	309.05	1.63	65	66	3.09 × 10^–6^			
	298.15	1.40	120	61	2.98 × 10^–7^			
acetonitrile	320.75	1.00	10	52	1.48 × 10^–5^	260.9	475.3	20.15
	318.95	0.96	21	51	8.49 × 10^–6^			
	316.65	0.90	46	50	4.13 × 10^–6^			
	314.05	0.85	78	48	1.80 × 10^–6^			
	305.65	0.68	251	44	1.13 × 10^–7^			
methanol	308.15	3.10	10	90	1.49 × 10^–7^	139.8	77.7	12.17
	303.15	2.90	17	87	5.97 × 10^–8^			
	301.15	2.82	29	86	4.10 × 10^–8^			
	298.15	2.70	54	84	2.32 × 10^–8^			
acetic acid	303.15	1.06	5	54	9.26 × 10^–10^	205.0	258.2	17.17
	298.15	0.94	21	51	2.33 × 10^–10^			
	293.15	0.83	26	48	5.58 × 10^–11^			

In the original papers on salicylic acid nucleation, rates for the nucleation from solutions in chloroform and acetone were included.^46,47,52^ Unfortunately, for chloroform the information on the temperature-dependent solution concentrations was insufficient to permit an Eyring–Polanyi analysis. For solutions in acetone, the Eyring–Polanyi analysis revealed that the temperature range over which the nucleation rate data were taken was so narrow that the solubility varied only from 2.59 mol L^–1^ to 2.39 mol L^–1^, i.e., by approximately 8%, as compared to ∼25%, ∼37%, ∼14% and ∼24% for ethyl acetate, acetonitrile, methanol and acetic acid, respectively. The extracted nucleation rate constants for nucleation from acetone were therefore, within the experimental error of the reported nucleation rates, too close to permit a meaningful linear regression analysis.

Nucleation rates predicted from the Δ*H* ^⧧^ and Δ*S* ^⧧^ values determined from the Eyring–Polanyi analysis are superimposed over the experimental nucleation rate data^47^ for the four solvents in Figure 6. All experimental data are reproduced well. The requirement of higher supersaturation in the order of ethyl acetate < acetonitrile < methanol < acetic acid is evident from the plots. What is also clear from these plots is that a minimum number of nucleation rate measurements over a judiciously chosen range of temperatures provides a quantitative understanding of both the supersaturation and the temperature dependence of the homogeneous nucleation rates. To provide a rule of thumb, temperatures should be chosen such that the underlying solubility variation is at least 10% to 15%. In the case of acetic acid, a total of three nucleation rate measurements over a solubility variation of ∼24% was sufficient to determine Δ*H* ^⧧^ and Δ*S* ^⧧^.

**Figure 6:**
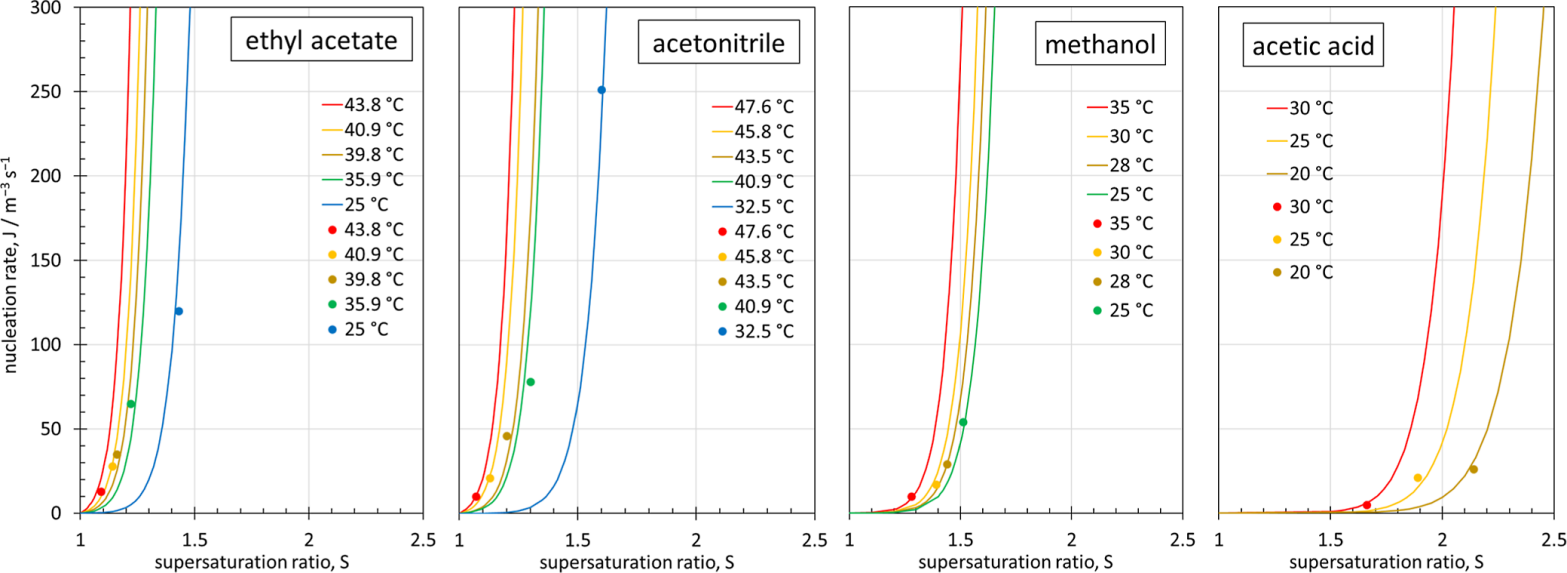
Symbols: temperature-dependent experimental homogeneous nucleation rates^47^ of salicylic acid in ethyl acetate, acetonitrile, methanol and acetic acid. Lines: nucleation rates predicted by eq 26 using the Δ*H* ^⧧^ and Δ*S* ^⧧^ values determined from the Eyring–Polanyi analysis in Figure 5.

## 5 Discussion

The Eyring–Polanyi analysis of the temperature-dependent nucleation rates data establishes that the nucleation rate constants exhibit Arrhenius behavior, indicating that the new homogeneous nucleation rate framework is consistent with fundamental rate theory. It is now worthwhile to examine the Δ*H* ^⧧^ and Δ*S* ^⧧^ values obtained from the Eyring–Polanyi analyses. Figure 7 contains what is often referred to as a “compensation plot” showing the activation entropy as a function of the activation enthalpy. A strong linear relationship between the two quantities is evident. In this case, it is an example for a trivial entropy–enthalpy compensation effect.^53,54^ It arises because the experimentally observable free energy activation barriers Δ*G* ^⧧^ for homogeneous nucleation of organic solutes from solution vary by only ±15% or so, as a consequence of the limited range of temperatures covered by the crystallization experiments. This limitation arises because measurement temperatures must be above the freezing point of the solvents, while an upper limit of accessible temperatures is determined by the vapor pressures of the solvents, which often become too high already at temperatures around ∼60 °C to ∼80 °C. The entropy–enthalpy compensation evident in Figure 7 is therefore not evidence for an isokinetic relationship^55,56^ or a kinetic compensation effect.^57,58^ It appears possible that more subtle entropy–enthalpy compensation effects may exist in relation to solvation,^59^ and with respect to the Barclay–Butler rule^60^ for the entropy of solution, but such links will have to be explored in future work.

**Figure 7:**
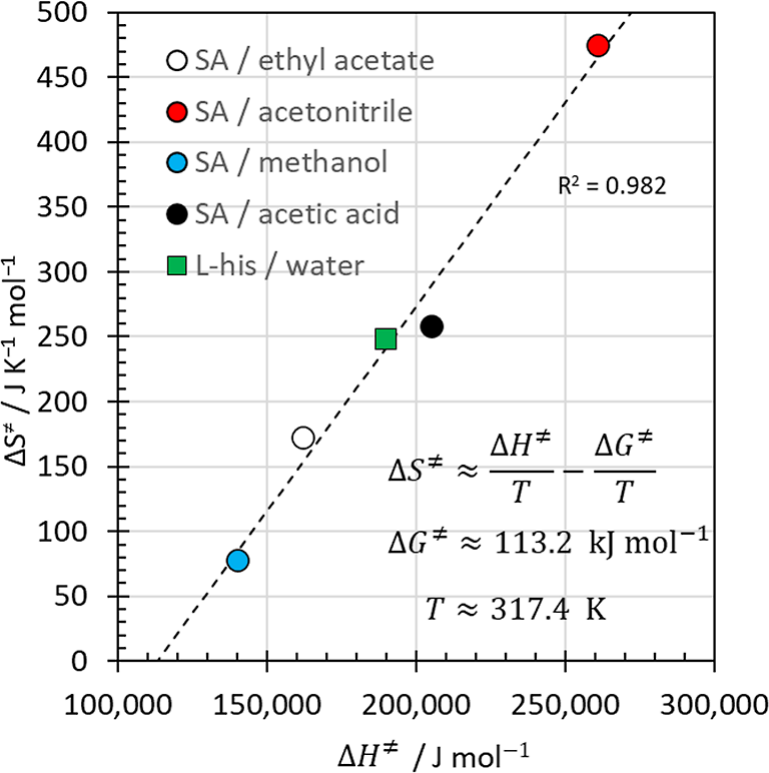
Compensation plot of activation entropies as a function of activation enthalpies for the homogeneous nucleation of l-histidine in water (Figure 4) as well as for salicylic acid in ethyl acetate, acetonitrile, methanol and acetic acid (Figure 5, Table 3). The linear regression line expresses the equation in the inset to the plot, corresponding to a free activation enthalpy Δ*G* ^⧧^ of 113.2 kJ mol^–1^ and a temperature of 317.4 K.

As shown in Figure 7, the linear regression fit represents a Gibbs free activation energy change Δ*G* ^⧧^ of 113.2 kJ mol^–1^ and a temperature of 317.4 K. Data points appearing above/below the line are associated with systems crystallizing above/below the temperature of 317.4 K. The salicylic acid/acetic acid system exhibits the strongest deviation because the nucleation rates require temperatures around 298 K, while all other systems crystallize with measurable rates at temperatures closer to 317.4 K (Table 3). For crystallization practice, the strong constraint on Δ*G* ^⧧^ imposed by the narrow range of experimentally accessible temperatures at pressures of 1 atm brings a practical benefit because it allows semiquantitative predictions of nucleation rates using eqs 21 and 24.

The extremely high temperature sensitivity of homogeneous crystal nucleation rates is accounted for through Δ*G* ^⧧^ activation barriers exceeding ∼100 kJ mol^–1^, which appear to be associated with multiexcitation events (vide supra)^48,49^ in forming the transition state. It is very interesting to note in this context that the activation enthalpies for the nucleation process scale, for all systems examined in this paper, linearly with the solution enthalpy (Figure 8), which is associated with dissolution, i.e., the reverse process to homogeneous nucleation. The strong correlation between the two quantities is suggestive of microscopic reversibility^61^ between nucleation and dissolution. Microscopic reversibility also provides a kinetic definition of solution saturation, namely the concentration for which the rate of crystal dissolution is equal to the rate of crystal nucleation proceeding through the largest LSDFs. In saturated solution, the size of these LSDFs is approximately n*, as given by eq 15, but their quantity, and hence the nucleation rate, is low. Such an integrated kinetic definition of solubility, dissolution and homogeneous nucleation would be consistent with a mechanistic view that dissolution and nucleation take place, at the molecular level, through the same sequence of bond-forming and bond-breaking events involving solute–solute and solute–solvent interactions. This concept would be interesting to explore further vis-a-vis the experimentally observable^20,21,23^ influence of thermal and other pretreatments of a solution on homogeneous nucleation phenomena. These would be difficult to explain by diffusion-driven short-lived solute density fluctuations and may point to the formation of additional metastable prenucleation clusters or phases.

**Figure 8:**
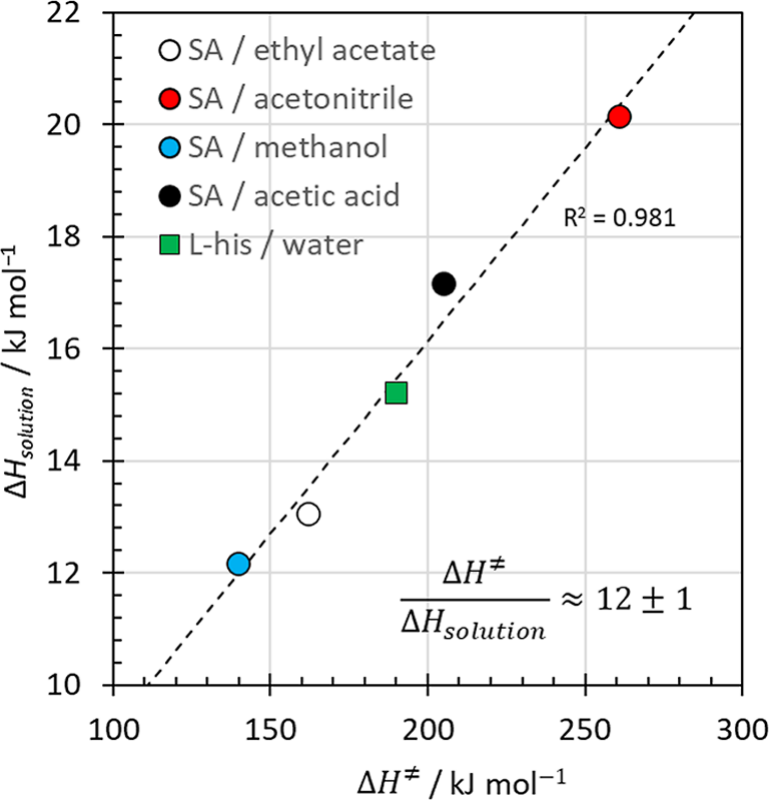
Linear correlation between the solution enthalpies and the determined activation enthalpies for homogeneous nucleation for l-histidine in water and for salicylic acid in ethyl acetate, acetonitrile, methanol and acetic acid.^46^ The linear regression fit indicates that the activation enthalpy is for all systems 12 ± 1 times the solution enthalpy.

Interestingly, the activation enthalpy for nucleation is for all examined systems approximately 12 ± 1 times the enthalpy of solution (Figure 8), implying that the transition state may, approximately, involve 12 ± 1 desolvated molecules. From a mechanistic point of view, this number is remarkably close to that of a densely packed cluster of spheres containing one central sphere surrounded by a coordination shell of 12 nearest neighbors. However, it is too early to assess whether an apparent universal “rule of 12 ± 1” between the enthalpies of dissolution and activation holds generally. It will need to be corroborated or refuted by future analysis of a wider range of systems. Nevertheless, the prospect of being able to predict the activation enthalpy from solubility measurements and estimate the activation entropy through a compensation plot such as that in Figure 7 is exciting. Such relations would be of extreme practical benefit for the design and control of crystallization processes.

Conceptually, the model of nucleation from diffusively driven excess LSDFs of solvated solutes is much more general than CNT (which could be seen as a special case of a very late transition state^12^) and shifts the focus from specific interfacial tension arguments toward a molecular-level mechanistic understanding of crystal nucleation via local density fluctuations. Very importantly the new model includes an explicit activation entropy contribution in the rate equation, as one would intuitively expect for a process involving desolvation (release of solvent molecules) and formation of an ordered crystal nucleus.

As previously noted,^12^ the nucleation from LSDFs is mechanistically not a two-step nucleation process along the lines widely discussed in the contemporary literature on nonclassical crystal nucleation pathways.^4,9,20,24–34^ LSDFs do not represent a separate intermediate phase; the diffusive motion of solvated solute molecules in a stable solution merely leads to short-lived spatial proximity between solvated solute molecules, on time scales of nanoseconds and with insufficiently attractive interactions to stabilize them against diffusive redissolution. The mechanism of homogeneous nucleation from LSDFs is also compatible with the enhancement of nucleation rates by heterogeneous nucleation in the presence of solid impurities or by seeding.^12^ However, the concentration of impurities required to achieve dominance of heterogeneous nucleation is likely higher than considerations based on CNT would suggest.^12^ Within the framework of nucleation from LSDFs, the rate of homogeneous nucleation events in a solution appear to dominate over heterogeneous nucleation events even when unfiltered high purity solvents are used.

Future work needs to examine how homogeneous nucleation from LSDFs can be integrated with the well-known sensitivity of homogeneous nucleation rates to agitation and shear.^62–64^ The size of the LSDFs is below the Kolmogorov limit where enhanced mass transport can be modeled by conventional Navier–Stokes fluid dynamics and may require more advanced molecular dynamics approaches.

## 6 Conclusions

The hypothesis that homogeneous organic crystal nucleation in solution is facilitated by LSDFs has been examined with a view to the temperature dependence of homogeneous nucleation rates. The temperature dependence was found to exhibit Arrhenius behavior and lends itself to an Eyring–Polanyi determination of activation enthalpies and entropies. The analysis of data for several systems indicates that the activation enthalpies relate to enthalpies of solution, which are readily accessible through temperature-dependent solubility measurements. Overall, a mechanistic picture of microscopic reversibility between dissolution and crystal nucleation in LSDFs appears to emerge.

Homogeneous small molecule nucleation appears to have similarities with molecular structure selection in biochemical systems^65^ in the sense that the activated complex represents a spatiotemporally highly specific multiexcitation event that leads to a product with highly specific composition and structure (here: a crystal structure). The magnitude of the activation parameters determined in this paper is comparable to those observed for protein denaturation/unfolding processes,^66,67^ which are similarly associated with multiple bond breaking and ordering events. The high substrate specificity afforded by macromolecular folding in biological transformations is another interesting parallel.^68^ Indeed, there is already evidence that specific prenucleation interactions between solute molecules may play a role in determining kinetically the polymorphic outcomes of small molecule crystallization events, aligning with facet-specific growth rates after the initial nuclei have been formed.^69–71^ Using eq 26, such enthalpic (and entropic) contributions associated with nucleus formation can now be linked quantitatively to nucleation rates, solution concentrations and temperatures.

To make progress with our understanding of the molecular basis for nucleation kinetics, more systematic and accurate temperature studies will be needed to determine Δ*H* ^⧧^ and Δ*S* ^⧧^ values for more systems. Determining activation entropy values requires particular care because the extrapolation to the intercept defined by eq 27 depends critically on the accuracy of the solubility and nucleation rate data, and more so than the determination of activation enthalpies from the slope of the Eyring–Polanyi plots. There are also many fundamental theoretical questions that require clarification. To give a few examples, the transmission coefficient κ is not known; proton tunnelling may have to be considered where proton transfer and/or breaking of hydrogen-bonds takes place;^39^ it is unclear whether the elementary steps in the nucleus assembly are sufficiently slow to justify an assumption that every intermediate formed in the sequence of elementary processes is long-lived enough to assume a Boltzmann energy distribution. The dynamics of the potential energy surface^65,72^ for nucleation need to be considered.

Mechanistically, the system-specific sequences of elementary events leading to nucleation in LSDFs are currently unknown. Studies of nucleation dynamics in dense, highly concentrated solutions, which have higher concentrations of LSDFs, may develop our understanding further. Unravelling ultrafast dynamics in the transition state may be required for understanding the relationships between the rate constant k* and the polymorphic outcome of nucleation events, including the formation of crystalline solvate structures.

## Supplementary Material



## Notes

Derivation of the equation for the size distribution of solute density fluctuations; derivation of the equation for the calculation of *n**; ratio between LSDF numbers for *K* ≠ 1 and for *K* = 1 for concentrations 0.01 to 5 mol dm^–3^ (PDF)
