# Supplementary material for: Eyring–Polanyi Rate Theory for the Homogeneous Nucleation of Organic Crystals from Solution
Source: Cryst Growth Des. 2025 Dec 12;26(1):19–31. doi: 10.1021/acs.cgd.5c00524 (PMC12784332; doi:10.1021/acs.cgd.5c00524)

Supplementary Information

Eyring-Polanyi Rate Theory for the Homogeneous Nucleation of Organic Crystals from Solution

Sven L. M. Schroeder

[1]School of Chemical and Process Engineering, University of Leeds, Leeds, LS2 9JT, UK

[2]Diamond Light Source, Harwell Science & Innovation Campus, Didcot, OX11 0DE, UK

[3]Future Continuous Manufacturing and Advanced Crystallisation (CMAC) Hub, Research Complex at Harwell (RCaH), Rutherford Appleton Laboratory, Didcot, OX11 0FA, UK

SI1 - Derivation of the equation for the size distribution of solute density fluctuations

Mass balance: overall activity a_0 of solutes and the individual activities a_n of solvated solute monomers (M) and dense regions M_n formed by diffusively driven collisions of the solvated solute monomers

$$a_0 = \sum_{n=1}^{\infty} n a_n$$

Equilibrium constant K_2 for the collision between two solute monomers, in terms of the activity of the formed solute dimer a_2 and the activity a_1 of monomeric solvated solute molecules:

$$K_2 = \frac{a_2}{a_1^2}$$

Equilibrium constant K_3 for the formation of a density fluctuation containing three solute monomers, introducing the activity of the density fluctuation a_3 and expressing it in terms of K_2

$$K_3 = \frac{a_3}{a_2 a_1} = \frac{a_3}{K_2 a_1^3}$$

Equilibrium constant K_4 for the formation of a density fluctuation containing four solute monomers, introducing the activity of the density fluctuation a_4 and expressing it in terms of K_2 and K_3

$$K_4 = \frac{a_4}{a_3 a_1} = \frac{a_4}{K_2 K_3 a_1^3}$$

General equilibrium constant K_n for the formation of a density fluctuation containing *n* solute monomers, introducing the activity of the density fluctuation a_n and expressing it in terms of the equilibrium constants $K_2 \ldots K_{n-1}$

$$K_n = \frac{a_n}{a_{n-1} a_1} = \frac{a_n}{(K_2 \times K_3 \times \ldots \times K_{n-1}) a_1^n}$$

Assume that K_n and all K_i in the denominator are approximately identical, which appears to be a good approximation especially as n increases, when each additional monomer to a larger dense region

$$K_2 \times K_3 \times \ldots \times K_n \approx K^{n-1}$$

We obtain

$$K^{n-1} \approx \frac{a_n}{a_1^n}$$

This can be rearranged to

$$a_n = a_1^n K^{n-1} = K^{-1} (K a_1)^n$$

which gives, with the mass balance (see the starting equation above)

$$a_0 = \sum_{n=1}^{\infty} n a_n \approx K^{-1} \sum_{n=1}^{\infty} n (K a_1)^n$$

Substituting $m = K a_1$, we can write

$$\mathrm{a}_0 K \approx \sum_{n=1}^{\infty} n m^n$$

The expression on the right is a power series with the solution

$$\sum_{n=1}^{k} n m^n = \mathrm{m}\frac{1-(k+1)m^k + k m^{k+1}}{(1-\mathrm{m})^2}$$

When $m = K\mathrm{a}_1 < 1$ (this condition will be fulfilled for almost all practical solutions, unless very high concentrations and/or large values of *K* due to very strong attractive solute-solute interactions apply), it follows that for $k \to \infty$,

$$m^k = 0$$

$$m^{k+1} = 0$$

Hence

$$\mathrm{a}_0 K \approx \frac{m}{(1-m)^2}$$

This is a 2nd order polynomial. Rearranged to the form $jx^2 + kx + l$ we get

$$m^2 - \left(2 + \frac{1}{\mathrm{a}_0 K}\right)m + 1 = 0$$

with

$$j = 1;\, k = -\left(2 + \frac{1}{\mathrm{a}_0 K}\right);\, l = 1$$

Solving with quadratic formula

$$\mathrm{m} = \frac{-\mathrm{k} \pm \sqrt{\mathrm{k}^2 - 4\mathrm{jl}}}{2\mathrm{j}} = \frac{\left(2 + \frac{1}{\mathrm{a}_0 \mathrm{K}}\right) \pm \sqrt{\left(2 + \frac{1}{\mathrm{a}_0 \mathrm{K}}\right)^2 - 4}}{2}$$

and some algebra gives

$$\mathrm{m} = \left(1 + \frac{1}{2\mathrm{a}_0 \mathrm{K}}\right) \pm \frac{1}{2\mathrm{a}_0 \mathrm{K}}\sqrt{4\mathrm{a}_0 \mathrm{K} + 1}$$

Only the subtraction gives a meaningful solution

$$\mathrm{m} = \left(1 + \frac{1}{2\mathrm{a}_0 \mathrm{K}}\right) - \frac{1}{2\mathrm{a}_0 \mathrm{K}}\sqrt{4\mathrm{a}_0 \mathrm{K} + 1}$$

Now multiply with $1 + \sqrt{4\mathrm{a}_0 K + 1}$ in the numerator and denominator:

$$\mathrm{m} = \frac{\left(\left(1 + \frac{1}{2\mathrm{a}_0 \mathrm{K}}\right) - \frac{1}{2\mathrm{a}_0 \mathrm{K}}\sqrt{4\mathrm{a}_0 \mathrm{K} + 1}\right)\left(1 + \sqrt{4\mathrm{a}_0 \mathrm{K} + 1}\right)}{1 + \sqrt{4\mathrm{a}_0 \mathrm{K} + 1}}$$

Algebra simplifies the numerator and we get

$$\mathrm{m} = \frac{\sqrt{4\mathrm{a}_0\mathrm{K}+1}-1}{\sqrt{4\mathrm{a}_0\mathrm{K}+1}+1}$$

Re-substituting $m = K\mathrm{a}_1$ we get the expression for the monomer activity as a function of overall solution activity and the equilibrium constant for solute-solute interactions

$$\mathrm{a}_1 = \frac{1}{K}\frac{\sqrt{4\mathrm{a}_0K+1}-1}{\sqrt{4\mathrm{a}_0K+1}+1}$$

An evaluation of this equation as a function of a_0 and K shows that for K = 0.001 (strong repulsive interactions corresponding to $-\mathrm{R}T\ln(K) = 17.2\ \mathrm{kJ\ mol^{-1}}$ at T = 300 K), practically all solvated solute molecules exist as monomers, for all solution activities a_0. For K = 1000 (strong solute-solute attraction with $-\mathrm{R}T\ln(K) = -17.2\ \mathrm{kJ\ mol^{-1}}$ at 300 K) monomer concentrations exceed 5% only at very low concentrations (for approximately $a_0 < 0.02$).

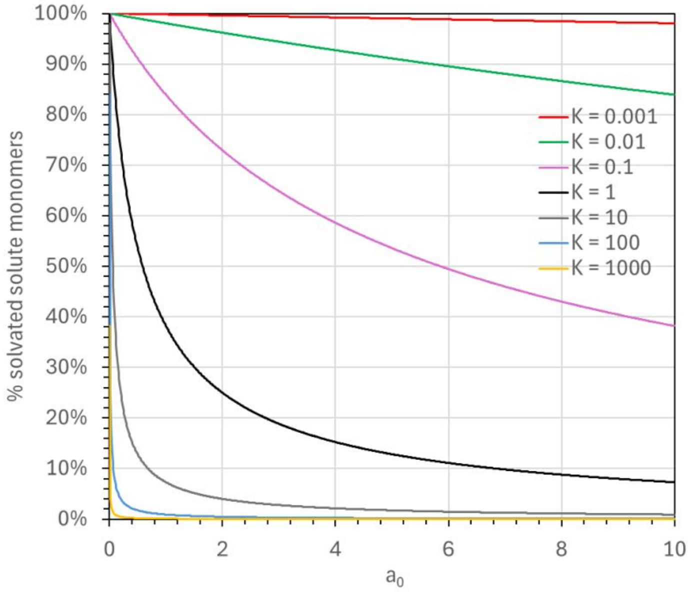

The final expression for the aggregate size distribution, using the earlier derived expression for $\mathrm{a_n}$ is:

$$\mathrm{a_n} = \frac{1}{K}(K\mathrm{a}_1)^n = \frac{1}{K}\left(\frac{\sqrt{4\mathrm{a}_0K+1}-1}{\sqrt{4\mathrm{a}_0K+1}+1}\right)^n$$

For an ideal solution we can replace the solution activity by the solute concentrations $[\mathrm{S_xM}]_0$ according to

$$a_0 = \frac{[\mathrm{S_xM}]_0}{c^{\ominus}}$$

With

$$c^{\ominus} = 1\ \frac{\mathrm{mol}}{\mathrm{dm^3}}$$

Giving

$$[(S_xM)_n] = \frac{1}{K}\left(\frac{\sqrt{4\frac{[S_xM]_0}{c^{\ominus}}K+1}-1}{\sqrt{4\frac{[S_xM]_0}{c^{\ominus}}K+1}+1}\right)^n$$

Numerically, since $c^{\ominus} = 1\,\frac{\text{mol}}{\text{dm}^3}$, the concentration $[(S_xM)_n]$ of any region with n solvated solute molecules in mol dm^{-3} is given by

$$[(S_xM)_n] = \frac{1}{K}\left(\frac{\sqrt{4K[S_xM]_0+1}-1}{\sqrt{4K[S_xM]_0+1}+1}\right)^n$$

Typical values of K can be estimated by considering that attractive interactions between solvated solute molecules must be weak enough to prevent an irreversible phase separation, e.g. by a liquid-liquid phase separation. For the diffusion-driven density fluctuations to be reversible attractive potentials are therefore likely to be less than a few k_BT. Expressing the driving force for attractive interactions as a percentage σ of k_BT predicts the equilibrium constants K as a function of σ.

$$K = \exp\left(-\frac{\Delta G}{RT}\right) = \exp\left(-\frac{-\sigma k_B T N_A}{RT}\right) = \exp(\sigma)$$

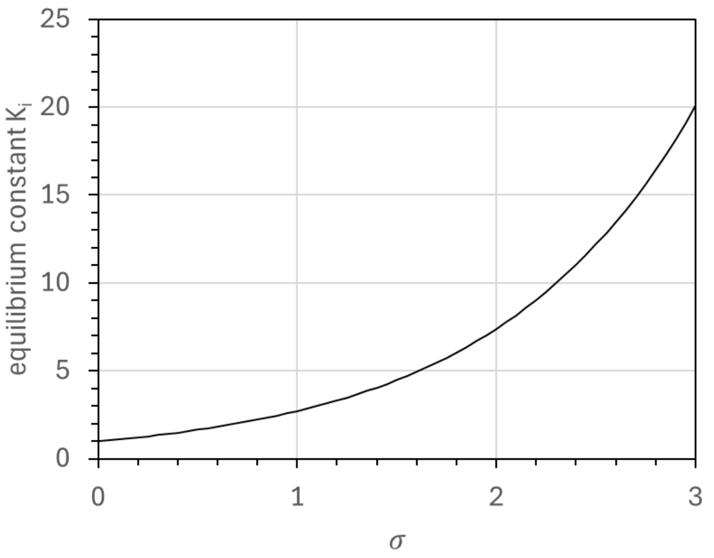

SI2 - Derivation of the equation for the calculation of n*

We derived in SI1 that

$$[(\mathrm{S_xM})_\mathrm{n}] = \frac{1}{K}\left(\frac{\sqrt{4K[\mathrm{S_xM}]_0 + 1} - 1}{\sqrt{4K[\mathrm{S_xM}]_0 + 1} + 1}\right)^n$$

At the solubility concentration $[\mathrm{S_xM}]_0^*$, the largest aggregate has a size of n*. There must be at least 1 of these aggregates, i.e.,

$$\mathrm{N_{n^*}} = \left[(\mathrm{S_xM})_{\mathrm{n}^*}\right] N_A V = \frac{1}{K}\left(\frac{\sqrt{4K[\mathrm{S_xM}]_0^* + 1} - 1}{\sqrt{4K[\mathrm{S_xM}]_0^* + 1} + 1}\right)^{\mathrm{n}^*} N_A V = 1$$

Solve for n*

$$\frac{1}{K}\left(\frac{\sqrt{4K[\mathrm{S_xM}]_0^* + 1} - 1}{\sqrt{4K[\mathrm{S_xM}]_0^* + 1} + 1}\right)^{\mathrm{n}^*} = \frac{1}{N_A V}$$

$$\mathrm{n}^*\ln\left(\frac{\sqrt{4K[\mathrm{S_xM}]_0^* + 1} - 1}{\sqrt{4K[\mathrm{S_xM}]_0^* + 1} + 1}\right) - \ln(K) = -\ln(N_A V)$$

$$\mathrm{n}^*\ln\left(\frac{\sqrt{4K[\mathrm{S_xM}]_0^* + 1} - 1}{\sqrt{4K[\mathrm{S_xM}]_0^* + 1} + 1}\right) = -\ln(N_A V) + \ln(K)$$

$$\mathrm{n}^* = -\frac{\ln(N_A V) - \ln(K)}{\ln\left(\frac{\sqrt{4K[\mathrm{S_xM}]_0^* + 1} - 1}{\sqrt{4K[\mathrm{S_xM}]_0^* + 1} + 1}\right)} = \frac{\ln(K) - \ln(N_A V)}{\ln\left(\frac{\sqrt{4K[\mathrm{S_xM}]_0^* + 1} - 1}{\sqrt{4K[\mathrm{S_xM}]_0^* + 1} + 1}\right)}$$

The resulting value for n* should be rounded down to the nearest integer.

SI3 – Ratio between LSDF numbers for K≠1 and for K=1 for concentrations 0.01…5 mol dm^{-3}

(a) For a solution volume V = 1 cm^3

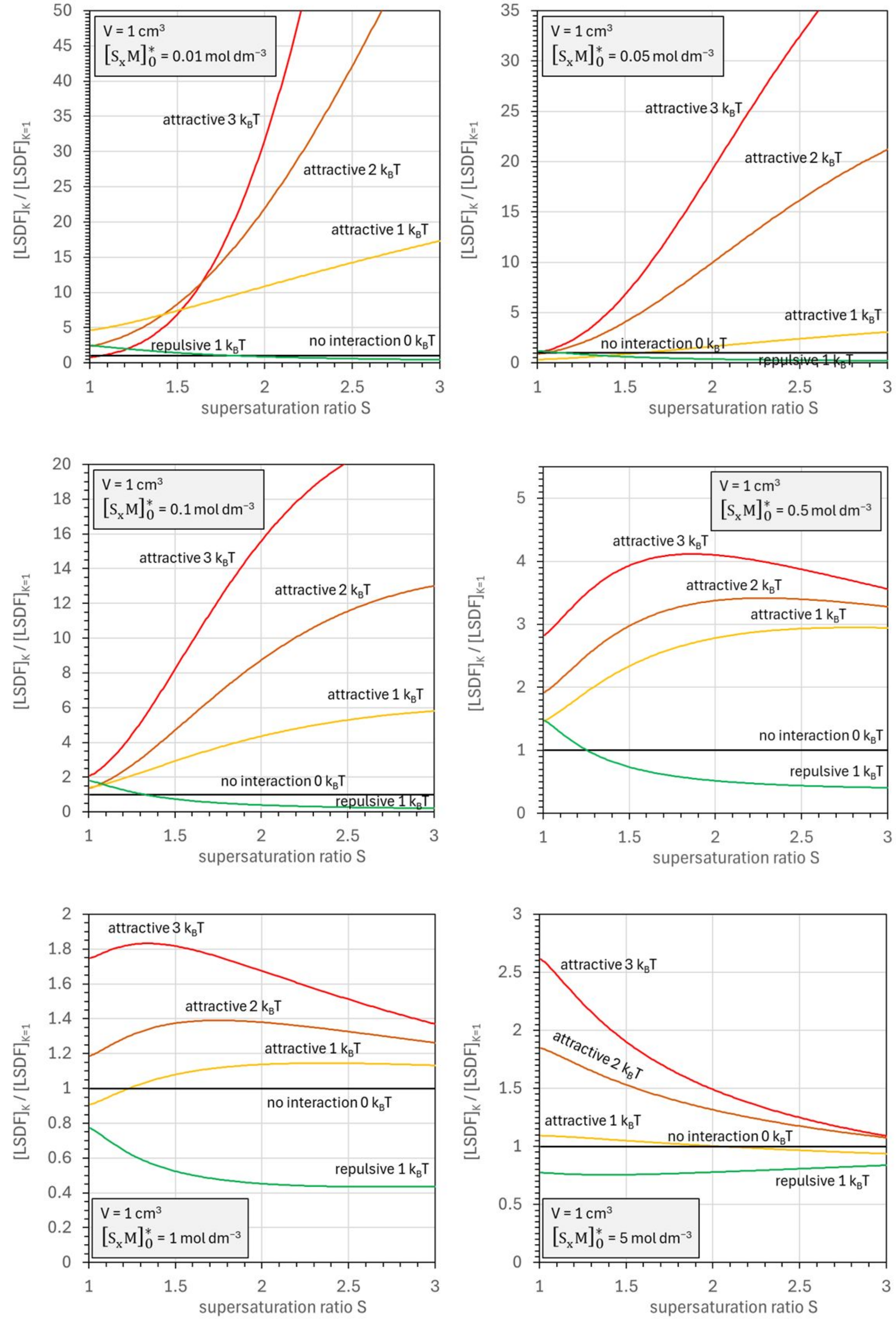

(b) For a solution volume V = 20 cm^3

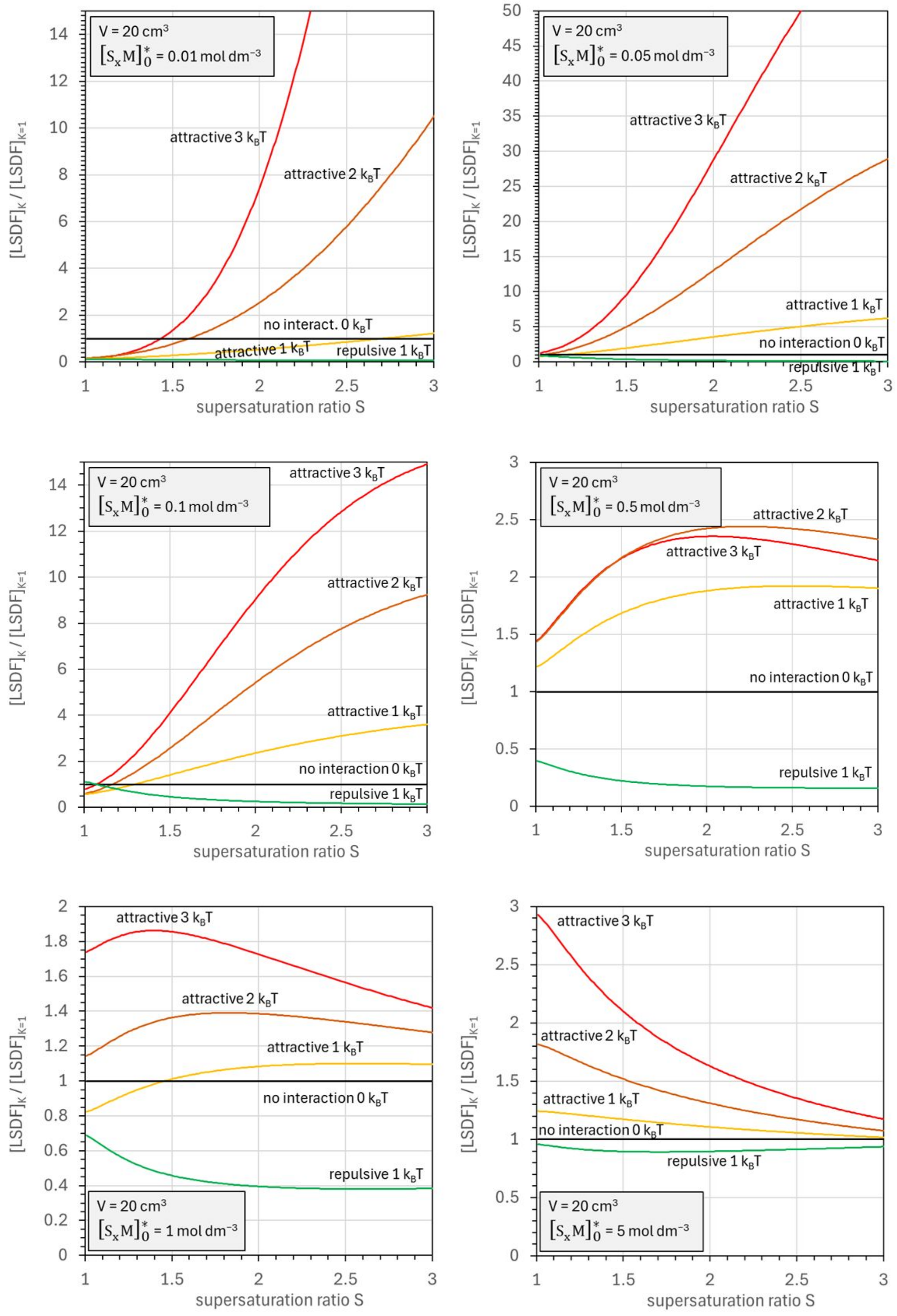

Supplement: Supplementary file 1 [file cg5c00524_si_001.pdf]
